# Exploring opportunities & pathways for advanced practice radiation therapy roles in the United States

**DOI:** 10.1016/j.tipsro.2021.01.005

**Published:** 2021-03-18

**Authors:** Samantha Skubish, Clodagh Starrs, Danielle McDonagh

**Affiliations:** a Department of Radiation Oncology, Mount Sinai Health System, 1184 5^th^ Avenue, New York, NY 10029, United States; b Department of Radiation Oncology, Mount Sinai Hospital, 1184 5^th^ Avenue, New York, NY 10029, United States; c Department of Radiation Oncology, Mount Sinai Beth Israel, 10 Union Square East, New York, NY 10003, United States

**Keywords:** Advanced practice radiation therapy, Evidence-based practice, Patient care, Radiation oncology, Innovative care models

## Abstract

The evolution of practice of Radiation Therapy in the United States (U.S.) is inevitable. The scope of a radiation therapists role has progressed with advancing technology, implementation of special procedures and patient care requirements. Internationally, Canada, Australia and the United Kingdom have formalized this evolution through the Advanced Practice Radiation Therapist (APRT) role to provide new models of care, to meet growing demands in the practice of Radiation Oncology, to increase efficiency, decrease cost and retain skilled staff (Harnett et al., 2018; Society of Radiographers; Linden et al., 2019; Coleman et al., 2014) [1], [2], [3], [4]. Through evidence based practice, the APRT role has proven to provide benefits for multiple stakeholders including service-reconfiguration to reduce wait times, developing and retaining highly skilled radiation therapists, treatment review and most importantly improving patient care within much needed patient cohorts such as the palliative population (Duffton et al., 2019) [5]. The U.S. is no exception to requiring innovative care models and solutions to similar complex, care delivery challenges experienced internationally. The U.S. is experiencing an increase in demand for cancer services and a rapid rate of technological and treatment advancements. Under the current infrastructure, this has impacted the daily tasks of physicians; increasing workload, increasing the complexity of clinical decision making, increasing movement toward site specific subspecialty practice and pushing the scope of radiation therapists informally toward maximization, increased autonomy and a higher level of education and specialized skills (American Society of Clinical Oncology, 2016; ARRT, 2020; Vu et al., 2018) [6], [7], [8].

**Objective**

The following reviews the current radiation therapy practice and professional landscape in the United States as it relates to advanced practice, exploring opportunities and challenges under the U.S. health care infrastructure. This broad analysis provides comparison to other countries and disciplines such as the Radiologist Assistant and Nurse Practitioner for potential pathways to establishing the role and describes current needs and value of the expanding scope of RT’s practicing in the U.S.

## Highlights

• Educational and regulatory frameworks do not exist for RTT scope expansion in the U.S.
• Institutions have tools through other AP roles and established international models.
• The APRT role could offer solutions to new pressures on the US health system.
• Institutional support, therapy initiated research and regulatory changes are required.
• Formal collaboration among institutions can revive efforts within the profession.

## Introduction to existing AP roles in the U.S.

Within the United States (U.S.) healthcare system, Advanced Practice models are widely utilized via Nurse Practitioners (NP’s) and Physician Assistants (PA’s) whom are often physician extenders to Radiation Oncologists in the clinic. The need for advanced practice nursing roles grew organically in the U.S. based on patient needs, system pressures and the nursing profession’s desire to practice in an expanded capacity. Barriers to implementation included outdated laws, resistance from physicians, lack of reimbursement policies by insurance companies and varying regulations state by state [9]. In 2019, the Centers for Medicaid and Medicare (CMS) expanded the scope of PA’s under new regulations, largely deferring to individual state laws and allowing more flexibility of duties [10]. This signals a growing level of support for AP roles within the U.S. payment infrastructure and perhaps a response to the anticipated physician shortages on top of an increase in sub-specialization [11]. These professions have experienced an evolving role in Oncology over the last several decades [11]. Notably, NP’s and PA’s within the Radiation Oncology department support clinic visits, symptom management and carryout administrative and research tasks [12]. In contrast, radiation therapists hold a distinct technical skill set required for an AP role to support patients daily through continuity of care and to support physicians in a different but equally important domain of daily Radiation Oncology practice such as with the ever expanding IGRT workload. It can be understood, that just as elevating nursing education has created successful AP nursing roles in the field, elevating therapists education can create the same level of support and outcomes with a distinct place for both in U.S. departments such as the treatment review clinics that are run by AP’s in international institutions.

Similarly, under the scope of technical professions and Radiologic Science, the Radiologist Assistant (RA) framework was established in 2003 as a physician extender to diagnostic Radiologists. RA’s complete an additional 2.5 years of graduate study and are trained to “perform selected radiology procedures, evaluate image quality and make initial observations” [13]. CMS recently changed supervision requirements for RA’s and demand for RA positions is said to be growing [13]. Although the model has historically struggled with reimbursement for RA specific activities, RA’s have proven to alleviate time for physicians to concentrate on reimbursable activities and can increase practice productivity, while enhancing patient satisfaction [14]. The RA role is representative of effective clinical role expansion in the radiologic sciences field in the U.S. and further modeling of advanced practice as a solution to health care delivery challenges. The APRT role could have similar benefits and there are lessons to be learned from the successes and challenges of establishing the RA role.

In 2007, Martino et al. completed an analysis and publication on establishing the APRT role in the U.S., defining territoriality among professions as a barrier to implementation [15]. However, the distinction between the skill set and motivations of RTT’s is starkly different from other disciplines within the radiation oncology professional sphere. Building upon the technical treatment and simulation expertise of the radiation therapist, and elevating that existing skill set, would avoid uneasiness with other disciplines. It would also offer opportunities on the professional promotion ladder that aligns with the therapist’s interests, an important issue of job satisfaction for a new generation of therapists advancing their careers. Learning from the NP, PA and RA experiences, the modern strategic approach must work toward a clearly defined role, address the unique health care infrastructure in the U.S. and elevate learned experiences in individual departments to enhance patient care.

## Discussion on APRT role

The Radiation Therapy workforce, a cohort of highly skilled radiation and patient care specifically trained personnel, has had an increasingly complex role among the multidisciplinary Radiation Oncology team [15]. The educational requirements for national certification have recently been elevated to require an Associate’s degree at a minimum in response to the growing skill set required in the profession, while the American Society for Radioloigc Technoloigsts (ASRT) has taken the position that a bachelors degree should be the required entry level education for Radiation Therapists [7], [16]. The motivations of therapists entering the field has also evolved. Just two decades ago, new therapists entering the technical field of Radiologic Science were likely motivated by job security, short educational programs, high demand and high pay. New therapists entering the field are highly motivated by patient care and the rapid advancements of technology, such as the wide adoption of proton therapy in the United States.

The U.S. credentialing organization for radiation therapists, the American Registry of Radiologic Technologists (ARRT), has addressed the unique education and competencies required for Proton Beam Radiation Therapy (PBRT). Under the ARRT, the Alternative Forms of Recognition (AFR) program was initiated to “provide a mechanism for individuals to document completion of activities that are prerequisite to the professional performance of a role in areas for which ARRT does not currently offer certification” [17]. A comprehensive list of knowledge requirements, practice analysis and task inventory was developed to represent the robust knowledge supporting the professional performance of a radiation therapist in PBRT. Pathways for candidates to achieve PBRT AFR have been identified and include evidence of clinical knowledge requirements [17]. The creation of AFR highlights the ARRT’s recognition of the advancements in radiation therapy as well as the body’s willingness to address the needs of the profession. Professionals in the field could interpret this program as the ARRT’s recognition of maximizing and expanding scope that may open a pathway to new clinical roles and new models of care, including advanced practice.

In 2004, the ASRT assembled a Radiation Therapy Clinical Practice Advisory Panel to consider establishing an APRT role in the U.S. [15]. The advisory panel concluded there were benefits to implementation however, the national conversation did not go much further beyond the publication of the white paper “Advanced Practice in Radiation Therapy” in 2007 [15]. A brief literature review shows very few publications in the United States regarding advanced practice radiation therapy have been published since [15], [18]. While many of the practitioners in the field are primed for such advancements, the lack of national conversation and support poses a huge barrier for long-term success. In an effort to “catch-up” with the countries that have been successful at establishing an APRT role, collaboration among academic institutions has the potential to revive the conversation and create momentum, while aiding in more quickly establishing the role.

The current U.S. health system infrasture has significantly changed since the publication of the white paper in 2007, with several key structural changes having occurred. Payment reforms such as the Alternative Payment Model has started a transition from a fee-for-service model toward bundled payments, decreasing the reimbursement paid per patient or treatment course overall [19]. This value-based model of care ultimately aims to drive care transformation while adding pressures on care delivery to be more efficient and of higher quality [19]. In doing so, fee-for-service reimbursable activities will matter less and efficiency and innovation will matter more. This structural change presents opportunity in determining the value of an advanced practice role in the U.S. despite some limitations on reimbursable activities. The additional financial hardship the Covid-19 pandemic has brought, further requires efficiency of care. Strategic planning has shifted from ensuring a steady growth in volume and revenue to recovery and innovations required to continue to offer high-quality care under mounting complexities and strains on the system [20]. In this context, the APRT role can be viewed as a solution to cost-effectiveness and to increase productivity under mounting financial pressures.

Furthermore, the educational infrastructure in the U.S. is bound to the scope of radiation therapists’ practice. This could be seen as a barrier to implementation. However, as practitioners, the RTT’s see patients more frequently than any other discipline, uniquely positioned to have the greatest impact on quality patient care. It would be logical to enhance the training, education and intellectual capital of this group to address gaps in care and inefficiencies [18]. When comparing to other countries, the structure and career path of the radiation therapist in the US is very different to the tiered approach in the United Kingdom (UK). In the U.S. there are limited pathways for the RTT beyond management, vendor employment, education or leaving the field entirely. In the UK, the established educational framework allows therapists to progress from novice to expert and to advance their clinical skills relative to their area of interest within a clear and concise scope of practice [21]. Through the four pillars of clinical practice, leadership, education and research, this educational model is often completed in tandem with daily practice in the clinic allowing service improvements and enhancements while continuing to prove the model and value of Advanced Practice [21].

Utilizing the UK perspective, a case study from the Radiation Oncology department at Mount Sinai Hospital in New York, NY describes the benefits experienced from AP education in application to daily operations. Uniquely positioned, the clinical manager, a Radiation Therapist, undertook a mentorship with an Attending Radiation Oncologist. They applied principles that were learned through the UK based Expert Practice Module from the MSc in Advanced Clinical Practice in Radiation Oncology program to a cohort of breast patients under the Attendings service. Although the intention was for the manager to progress from novice to expert in image review for the Masters program, the application of the four pillars of advanced practice provided a much greater benefit to the department than initially expected. As a direct response to the advanced practice module, the implementation of informed huddles with the physician was added to the therapist simulation workflow. This resulted in less manipulation of beams during the planning process, enhancing quality and efficiency by minimizing the verification appointment duration, subsequently improving patient experience. Through an enhanced focus on multidisciplinary communication, a more customized clinical approach was experienced for each patient. The entire Simulation team benefitted from a deeper understanding of physician clinical decision making regarding beam arrangement, patent’s history, surgical techniques, histology and tumor grade. The most notable improvement was a substantial decrease in image rejections over a three-month period preceding this project within this specific service [22].

The momentum and adoption of an APRT role will largely depend on the extent to which such efforts as those laid out in this case study are formalized by practicing RT’s and supported in individual departments by clinical management, administration and physicians. Trailblazing the APRT role will also require similar educational structure that is tailored to meet the unique needs of care delivery in the U.S. as well as evidence-based practice and research to drive innovation, steer the pathway of the profession and establish long-term value and sustainability of role.

Research has been estabilished as an integral part of the four pillars of advanced practice. It provides the reasoning for progression in practice of Radiation Therapy and must uphold the rigorous quality, and ethical obligations required by scientific discovery [23]. Radiation therapists in the U.S. may or may not be aware of the evidence-based practice that exists in Canada, the UK, Australia, and New Zealand [24], [25], [26]. The best pathway for instituting similar efforts in AP design is to begin with surveys and cohort studies on topics such as perception, gaps, education, and barriers while studying the task dileniation that would assist in a higher quality more efficient patient care process. Unfortunately, very few radiation therapists in the U.S. participate in research due to staffing levels recommended by governing bodies and a push toward lean operations. There must be a concerted effort to support therapy initiated research in the U.S. in order for an APRT pathway to exist.

Palliative radiation therapy presents a growing need in the U.S. and across the globe the APRT role continues to provide mechanisms and resources to address gaps in palliative care [27]. Cancer incidence in the U.S. will increase more than 45% over the next decade and more than half of those cases are palliative in nature, requiring some form of radiation therapy [28]. The burden of palliative care in Radiation Oncology continues to grow with the demand for patient-centered-care requiring physicians to address a gamut of needs - according to Parker et al., “physical symptoms, psychosocial issues, cultural considerations, spiritual needs, care coordination, advance care planning, goals of care, and ethical and legal issues” [28]. Despite the growing number of patients and their mounting concerns, there is a lack of education, emphasis, and research on how radiation therapists can fill this gap. There is growing effort toward subspecialized residents, oncologists, and fellows with specific knowledge of how to treat palliative patients [29]. Even so, it is well documented that a multidisciplinary and interdisciplinary approach would be a better to mitigate and improve patient outcomes [28], [29]. An advanced practice radiation therapist role would enable therapists to be a multidisciplinary team member to support these unique needs, due to the unique knowledge of daily patient interactions, observed pain, and how to better care for these patients on the treatment floor. Consequently, by employing research as the tool to develop the APRT role and then participate in research clinically, U.S. radiation therapists could be on par with the rest of the global communities.

## Conclusion

A systematic evidence-based approach in establishing the APRT role in the U.S. should be viewed as a solution to the growing complexity of the field of Radiation Oncology and the unique pressures of the U.S. health care system, including bundled payments, mounting financial pressures on Radiation Oncology departments and increased physician workload. While those practicing radiation therapy are elevated to new clinical and educational standards and look for clinical opportunities on the professional advancement ladder, there is no regulatory, educational or structural framework that offers a pathway for expanding the clinical role of the radiation therapist in the U.S.. However, institutions have the tools to establish pathways for APRT by learning from other AP roles, well established international models and through therapy initiated evidence-based research. Such efforts will help provide a pathway specific to the needs in the U.S. through associated savings, efficiencies, task dileniation and quality enhancements, especially for acute patient populations such as the palliative patient population. While pockets of interest and conversations about APRT have been a part of the profession for over a decade, formal collaboration is needed to revive and formalize efforts. Academic partnerships will help support the educational and clinical research components required for next steps and will aid in quickly adopting and establishing the role and educational infrastructure. At the instituoinal level, buy-in will largely depend on the advocacy of those working in the profession. Learning from the experiences of NP, PA and RA professions, institutions interested in exploring an APRT role should conduct a thorough review of local state practice guidelines and the legislative process for expansion. Nationally, professional institutions and accrediting bodies such as the ASRT and ARRT must recognize that there is a significant need for clinical career advancement opportunities at this point in the evolution of the scope of practice and prioritize the radiation therapy profession beyond current efforts. Grants and advocacy must support therapy driven research, as the APRT role cannot begin to exist without this. Building a foundation through initiatives discussed above, will justify the value of the APRT role: expanding the scope of the radiation therapist, strategically minimizing costs while improving patient care.

## Declaration of Competing Interest

The authors declare that they have no known competing financial interests or personal relationships that could have appeared to influence the work reported in this paper.
